# Autologous and Nonautologous Blood Transfusion in Patients with Ruptured Ectopic Pregnancy and Severe Blood Loss

**DOI:** 10.1155/2017/7501807

**Published:** 2017-06-14

**Authors:** Jingxian Huang, Dongquan Qin, Chunlin Gu, Yanjuan Huang, He Ma, Huageng Huang, Fanke Huang, Jiaxin Ruan, Mei Ling

**Affiliations:** Department of Anesthesiology, The Third Affiliated Hospital of Guangxi Medical University, Nanning, Guangxi 530031, China

## Abstract

**Background:**

There are some theoretical concerns for the use of intraoperative cell salvage (ICS) in patients with ectopic pregnancy. This study aimed to observe the impact of ICS on the coagulation function and clinical outcomes of patients with ruptured ectopic pregnancy and severe blood loss.

**Methods:**

This was a retrospective study of 225 patients with ruptured ectopic pregnancy and severe blood loss treated at the Third Affiliated Hospital of Guangxi Medical University between January 2012 and May 2016. Patients were grouped according to ICS (*n* = 116) and controls (*n* = 109, allogenic transfusion and no transfusion).

**Results:**

Compared with controls, patients with ICS had shorter hospitalization (*P* = 0.007), lower requirement for allogenic blood products (*P* < 0.001), and higher hemoglobin levels at discharge (*P* < 0.001). There were no complications/ adverse reactions. In the ICS group, hemoglobin at discharge (−6.5%, *P* = 0.002) and thrombin time (−3.7%, *P* = 0.002) were decreased 24 h after surgery, while 24 h APTT was increased (+4.6%, *P* < 0.001). In the control group, hemoglobin at discharge (−16.8%, *P* < 0.001) was decreased after surgery and 24 h APTT was increased (+2.4%, *P* = 0.045). At discharge, hemoglobin levels were higher in the ICS group (*P* < 0.001).

**Conclusion:**

ICS was associated with good clinical outcomes in patients with ruptured ectopic pregnancy and severe blood loss.

## 1. Introduction

Intraoperative autologous transfusion is widely used to retransfuse back the patient's own blood [1]. Three techniques are used: (1) intraoperative cell salvage (ICS) (blood is collected during surgery, filtered, washed, and transfused back); (2) preoperative autologous donation (blood is collected and stored before surgery); and (3) acute normovolemic hemodilution (ANH) (blood is collected immediately before surgery, blood volume is restored using fluids, and the blood is reinfused during surgery after major blood loss has ceased, or sooner if indicated). Autologous transfusion is in contrast with allogenic blood transfusion, for which the blood comes from an unrelated or anonymous donor [2]. The main factors in favor of autologous transfusion are the reduction of the risk of blood-borne infections (HIV, hepatitis, prions, etc.) and the protection of a scarce resource [2, 3].

Currently, the most commonly used method is cell salvage [2]. After washing, the autologous blood mainly includes packed red blood cells and coagulation components such as plasma, platelets, and coagulation factors have been mostly removed, thus having an adverse effect on the coagulation function of the patients [4, 5]. Therefore, it is suggested to add coagulation factors to the transfused blood, but doing so raises the costs of the procedure and increases the risk of diseases, especially if blood products are used [2]. Nevertheless, some North American and European studies have demonstrated that normal blood coagulation can be achieved if the concentration of coagulation factors in the transfused blood can be maintained at 20–30% of the normal levels, without the use of coagulation factors [6]. Coagulopathy may be considered when the volume of transfused blood is >2 L and coagulation function tests should then be performed, but there is no recommendation for the use of coagulation factors in all cases [7].

In addition, there are theoretical concerns when autologous transfusion is used in patients with ectopic pregnancy and severe blood loss, because the recovered blood is mixed with amniotic fluid and fetal blood, which may cause iatrogenic amniotic fluid embolism and alloimmune hemolysis [8, 9].

Therefore, the present study aimed to observe the coagulation function and clinical outcomes of 116 patients with ectopic pregnancy and severe blood loss treated with autologous transfusion. These patients were compared with patients who received allogenic transfusion. The results could provide evidence for the management of blood transfusion in patients with ectopic pregnancy and severe blood loss.

## 2. Methods

### 2.1. Study Design and Patients

This was a retrospective study of prospectively collected data of consecutively enrolled patients with ruptured ectopic pregnancy and severe blood loss treated at the Third Affiliated Hospital of Guangxi Medical University between January 2012 and May 2016. Starting in July 2013, cell salvage transfusion was used in patients with ectopic pregnancy and severe blood loss, defining a blood loss of >30% of total blood volume [10].

Ectopic pregnancy was diagnosed based on medical history and imaging. Using the database, the inclusion criteria were (1) ectopic pregnancy and (2) acute blood loss accounting for >30% of total blood volume, which is a nonmandatory indication for transfusion therapy [10]. The exclusion criteria were (1) use of any drugs affecting the coagulation function; (2) primary blood diseases; (3) ischemic heart disease; (4) requirement for cardiopulmonary resuscitation due to severe hemorrhagic shock; or (5) patients with concurrent intrauterine pregnancy and ectopic pregnancy.

Written informed consent was obtained from all patients included in the study and the ethics committee of the Third Affiliated Hospital of Guangxi Medical University approved the study.

### 2.2. Grouping

During the study period, there were 225 patients with ruptured ectopic pregnancy and acute blood loss. Patients were grouped according to autologous blood transfusion (*n* = 116) and controls (*n* = 109, including patients with allogenic transfusion and patients without transfusion).

Each group was divided into subgroups based on the rule that 10% increase (400 mL) of blood loss was considered to be a group: blood loss in the N1, N2, N3, N4, N5, and N6 subgroups was 1200–1599 mL, 1600–1999 mL, 2000–2399 mL, 2400–2799 mL, 2800–3199 mL, and ≥3200 mL, respectively. The basis for selecting these subgroups was that, in the present study, the mean weight of the patients was 50 kg and their mean blood volume was 4000 mL (50 kg × 8% [10]). An acute blood loss of 20–30% of the blood volume will result in a shock state and blood transfusion therapy can be considered if there is an acute blood loss of >30% (i.e., 1200 mL) [10]. Therefore, we used a blood loss of 10% of the blood volume (i.e., 400 mL) to divide the subgroups as above.

### 2.3. Surgical Approach

The surgical approach was recommended by the attending physician according to the patients' specific conditions and the final decisions were made by the patients. Most surgeries (94.2%) were laparoscopic surgeries; laparotomies were performed because of patients' will or critical condition that immediate surgery was needed. Physicians were more conservative during the January 2012 to January 2013 period, and there were slightly more laparotomies during that period.

Debridement of ectopic pregnancy was performed in all patients. If it was found during the operation that the patients had other diseases that required surgery, the appropriate procedures were undertaken at the same time. Therefore, some patients also received myomectomy/ovarian cystectomy/tubal repair and orthopedic/bilateral tubal ligation. The surgeries were not performed by the same surgeon, but all procedures were performed by operators with surgical qualification.

Intravenous general anesthesia was used, and the narcotic drugs included midazolam, fentanyl, remifentanil, propofol, and cisatracurium besylate. In the perioperative period, limited fluid resuscitation was used, and the major crystalloid solution was sodium lactated Ringer's solution (Sichuan Kelun Pharmaceutical Co., Ltd., China), while hydroxyethyl starch 200/0.5 and physiological saline (Hangzhou Minsheng Pharmaceutical Co., Ltd., China) were used for fluid expansion. If blood pressure was <90/60 mmHg, vasopressors such as ephedrine, dopamine, and norepinephrine were used to maintain blood pressure at a normal level.

### 2.4. Cell Salvage Transfusion

The cell salvage device was an autologous-P3000 blood recovery machine (Beijing Jingjing, Beijing, China). Matching double-lumen suction lines were used to retrieve the blood in the peritoneum. The recovered blood was mixed with heparin (12,500 U mixed with 500 mL of 0.9% sodium chloride) at 200 U of heparin for 100 mL of blood. Blood was placed into the centrifugal tank for centrifugation and washing. It was pumped into blood recovery bags before being transfused to the patients. The washing solution was 0.9% sodium chloride and 1000 mL was usually needed to wash 300 mL of red blood cells.

A restrictive transfusion strategy was used in all patients. Based on guidelines for perioperative transfusion and adjuvant therapy published by the American Society of Anesthesiologists in 2006 and the guidelines for AABB transfusion in the United States in 2012, red blood cells were infused if hemoglobin levels were <60–70 g/L, and transfusion was not needed if hemoglobin levels were >100 g/L [11, 12]. For patients with hemoglobin levels 60–100 g/L, whether transfusion was conducted or not was determined by the physicians according to comprehensive factors of the degree of anemia, cardiopulmonary decompensation function, metabolic rate, and age [11, 12]. Starting on July 2013, ICS was used for patients with ectopic pregnancy, and its use was based on the recommendation of the physician and the willingness of the patients.

Coagulation function was monitored according to guidelines [11], using standard laboratory diagnostic tests such as prothrombin time (PT), activated partial thromboplastin time (APTT), thrombin time (TT), and fibrinogen levels. Blood cells were analyzed using a Sysmex® XN-9000 Automated Hematology Systems (Sysmex, Kobe, Japan). A STA-R evolution automated coagulation device (Stago, Paris, France) was used to measure the coagulation indexes.

Before any surgery with the possibility of a transfusion, the patients or their legal representative was fully informed about the possibility of a transfusion, benefits, risks, and alternative options. The patients or their legal representative signed an informed consent.

In addition to transfusion therapy, patients with postoperative hemoglobin levels of 60–80 g/L received auxiliary method of intravenous iron supplement for about 4 days after the operation.

### 2.5. Data Collection

PT, APTT, international normalized ratio (INR), thrombin time (TT), and fibrinogen levels were collected. Clinical indicators included cure rate, ICU occupancy rate, classification of wound healing, hospitalization duration, operation time, surgery-related complications (postoperative infection and bleeding), amount of blood loss, amount of homologous transfusion/autologous transfusion during hospitalization, transfusion rate, adverse transfusion reactions (allergy, purpura, nonhemolytic febrile reaction, bleeding, and hemolysis), and anemia-related complications (dizziness, palpitation, chest distress, and chest pain) were also collected.

Calculation of the amount of blood loss included the amount of autologous retrieved blood, suction bottles, the sum of intraoperative blood clots, and pads. The calculation of blood clots and pads was based on empirical estimation. The operation time was from the time of surgical incision to the time of surgical suture. The patients were considered cured when their general state was good, vital signs were normal, and the concentration of serum human chorionic gonadotropin was decreased to normal.

### 2.6. Follow-Up after Discharge

The patients were considered as cured when their general condition was good, with normal vital signs, primary healing of the surgical incisions, and a decline to normal serum levels of HCG. All patients were required to have routine telephone follow-up within 2 weeks after discharge. Patients were required to pay a regular outpatient visit to check the HCG levels. Normally, the HCG levels should show a declining trend until normal. If the HCG levels keep increasing instead of declining and an abnormal HCG result is detected within 2 months postoperatively, rehospitalization for further examination and treatment is required. In the present study, no patients need to return to the hospital because of abnormal HCG results after discharge and there was no patient lost to follow-up.

### 2.7. Statistical Analysis

Normally distributed continuous data were presented as mean ± standard deviation and analyzed using Student's *t*-test. Nonnormally distributed continuous data were presented as median (range) and analyzed using the Mann–Whitney test (unpaired data) or Wilcoxon's paired signed rank test (paired data). Categorical data were presented as proportions and analyzed using the Chi-square test. SPSS20.1 (IBM, Armonk, NY, USA) was used for data processing and analysis. Two-sided *P* values <0.05 were considered statistically significant.

## 3. Results

### 3.1. Characteristics of the Patients

Table 1 presents the characteristics of the patients. All characteristics were similar between the two groups, except the surgical approach (*P* = 0.007), which was because the laparotomy approach and nonautologous transfusion were preferred prior to 2013.

### 3.2. Operative Characteristics

Table 2 presents the operative characteristics of the patients. Compared with the control group, patients in the ICS group had a slightly shorter hospitalization (*P* = 0.007), lower requirement for allogenic blood products (*P* < 0.001), and higher hemoglobin levels at discharge (*P* < 0.001). There were no complications or adverse reactions in the two groups.

### 3.3. Coagulation Function

Table 3 presents the coagulation function of the patients. Before surgery, thrombin time was higher in the ICS group (*P* < 0.001). In the ICS group, hemoglobin (−6.5%, *P* = 0.002) and thrombin time (−3.7%, *P* = 0.002) were decreased after surgery, while APTT was increased (+4.6%, *P* < 0.001). In the control group, hemoglobin (−16.8%, *P* < 0.001) was decreased after surgery and APTT was increased (+2.4%, *P* = 0.045). After surgery, hemoglobin levels were higher in the ICS group (*P* < 0.001).

### 3.4. Subgroup Analysis

**Figure 1:**
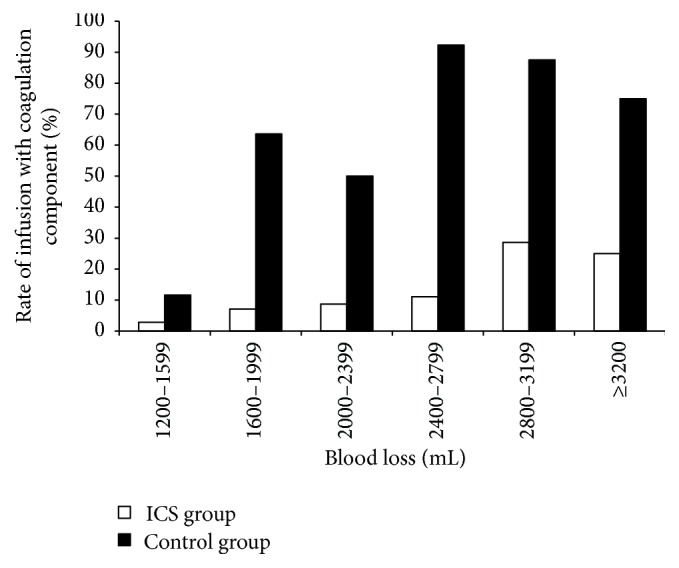
Rate of infusion of coagulation component (%) in the ICS and control groups.

Table 4 presents the characteristics of the patients according to the amount of blood loss.  Figure 1 shows that the rate of infusion of coagulation components was constantly higher in the control group than in the ICS group across all blood loss subgroups. These data could explain the differences in the coagulation functions according to blood loss, as presented in Table 5. Accordingly, postsurgical PT (median, 14.7 versus 13.9 s, *P* = 0.03) and INR (median, 1.14 versus 1.07 s, *P* = 0.009) were higher in ICS patients in the 1200–1599-mL blood loss subgroup. Pre- and postsurgical thrombin time were higher in ICS patients in the 2000–2399-mL-blood loss subgroup (before: median, 16.2 versus 14.1 s, *P* < 0.001; after: median, 16.3 versus 15.2, *P* = 0.001). In the ICS group, the 1200–1599-mL blood loss subgroup showed improvements in PT-INR (median, from 1.07 to 1.14, *P* = 0.032) and APTT (median, from 33.7 to 35.8, *P* = 0.017) after transfusion. In the control group, the 1600–1999-mL blood loss subgroup showed improvements in APTT (median, from 33.2 to 36.2, *P* = 0.050) after transfusion.

## 4. Discussion

With the increasing demand for blood transfusion, there are shortages of allogenic blood resources. In addition, there are risks with allogenic transfusion, such as transfusion reactions, infectious diseases, increased mortality, organ dysfunction, and delayed wound healing [2]. There are some theoretical concerns for the use of cell salvage transfusion in patients with ectopic pregnancy and severe blood loss. Therefore, the present study aimed to observe the impact of cell salvage transfusion on the coagulation function and clinical outcomes of patients with ruptured ectopic pregnancy and severe blood loss. Results suggest that ICS was associated with good clinical outcomes in patients with ruptured ectopic pregnancy and severe blood loss.

In the present study, the amount of blood loss in the ICS group was about 2300 mL. There were only nine patients who required allogenic blood products, accounting for 7.7% of the patients, and the average amount was 400 mL. One patient had a 4000-mL blood loss (>90% of the estimated blood volume) and received an autologous transfusion of 2500 mL, 400 mL of plasmas, and 10 U of cryoprecipitate. In the present study, blood loss was similar between the two groups.

Howard [4] reported that, through repeated washing, cell salvage transfusion can lead to a lack of coagulation factors, potentially leading to coagulopathy and even serious complications. Rollins et al. [5] reported one patient who received complex aortic surgery under cardiopulmonary bypass: the patient received a large amount of autologous transfusion in the operation and postoperative coagulopathy occurred; the state of the patient improved after using protamine to neutralize heparin.

North American and European studies have shown that normal blood coagulation can be achieved after cell salvage transfusion if the coagulation factors can be maintained at 20–30% of the normal levels [6]. Retrospective and prospective cohort studies, mostly performed in military trauma patients, suggested that early fresh frozen plasma transfusion with fresh frozen plasma to packed red blood cells ratio between 1 : 2 and 1 : 1 reduces 30-day mortality [13]. However, the evidence for this is of low quality and there is a lack of prospective randomized trials [13].

Ruptured ectopic pregnancy is a common gynecological problem and the patients suffer from hemorrhagic shock due to blood loss. Thus, emergency surgeries are needed. The condition of these patients is acute, dangerous, and severe, and they often require transfusion therapy. ICS transfusion could be used in ruptured ectopic pregnancy bleeding, but there is a theoretical risk of iatrogenic amniotic fluid embolism [8, 9]. In this study, no such event was observed. Clark et al. [14] suggested that amniotic fluid is already present in normal pregnancy maternal blood and that the amount of amniotic fluid in the blood recovered after cesarean section and was lower than that of the maternal blood itself. Morikawa et al. [15] reported a study of 50 patients, in which ICS transfusion was used for hemorrhage after cesarean section; 27 patients received preoperative stored autologous transfusion at the same time. The maximum amount of recovered blood in the operation was 3715 mL, and all patients had no transfusion-related adverse reactions or complications due to iatrogenic amniotic fluid embolism. The National Institute for Clinical Excellence (in Britain) had recommended using leukocyte depletion filter. Many previous studies had confirmed that the application of leukocyte depletion filter could further reduce the level of amniotic fluid components in the recycled blood [16]. However, the leukocyte depletion filters could induce hypotension; thus, the prognosis of the patients may also be influenced [17]. Therefore, the physicians should be particularly cautious. The specific reason might be that the removal of leukocytes resulted in the decrease of cytokines level and the increase in the release of vasodilator substances such as bradykinin and so on [18]. A review by Tevet et al. [19] strongly suggests that the use of ICS transfusion during the perinatal period was safe. Nevertheless, there is also a theoretical risk when the mother's Rhesus is negative and that of the fetus is positive. In this study, there were no Rhesus negative women, preventing any analysis of this point. Ralph et al. [20] suggested that fetal blood may enter into the mother during pregnancy and delivery but that autologous blood containing some fetal red blood cells had no significant adverse impact on the mother. When maternal blood is Rhesus negative, the guidelines of the British Committee for Standards in Hematology (BCSH) recommend that, after infusing autologous blood, the mother should receive an intramuscular injection of anti-D immunoglobulin to prevent fetal hemolytic disease [21].

In the present study, all clinical outcomes were similar between the two groups, including cure rate, ICU occupancy, operation duration, wound healing, and complications. Hospitalization duration was significantly shorter in the ICS group, suggesting that ICS had good clinical outcomes. Priuli et al. [22] reported that, in West African countries where blood stores are minimal, cell salvage transfusion led to no adverse reactions and complications and that postoperative recovery was good. Selo-Ojeme and Feyi-Waboso [23] reported that there were no differences in the occurrence of postoperative fever and postoperative wound infection between autologous and allogenic blood transfusion and that hospitalization duration was shorter with ICS. Uchil reported that the use of ICS during cesarean section decreased the requirement for allogenic transfusion, without adverse effects [24].

The present study is not without limitations. In the calculation of blood loss, the calculation of blood clots and cotton pads was based on an empirical estimation. The selection of the surgical approach had a bias during the early stage of the study period and some patients underwent multiple procedures at the same time, which would impact operation time and hospitalization days. In the early stage of the study period, under the influence of the traditional concept that plasma infusion for acute patients with severe blood loss is helpful to improve prognosis, some physicians failed to strictly enforce the indications for plasma transfusion in the nonautologous transfusion group, which resulted in unnecessary transfusion and waste of blood products and increased the risk of allogenic transfusion. Additional studies are still necessary to confirm the safety and efficacy of ICS for patients with ruptured ectopic pregnancy and sever blood loss.

## 5. Conclusions

ICS was associated with good clinical outcomes in patients with ruptured ectopic pregnancy and severe blood loss. These results provide some evidence for the management of blood transfusion in patients with ectopic pregnancy and severe blood loss.

## Figures and Tables

**Table 1:** Baseline characteristics of the patients.

	ICS (*n* = 116)	Controls (*n* = 109)	*P*
Age (years)	30 (17, 48)	32 (18, 45)	0.162
Weight (kg)	50 (40, 75)	50 (32, 80)	0.014
Surgical approach			0.007
Laparotomy	2 (1.72%)	11 (10.09%)	
Laparoscopic	114 (98.28%)	98 (89.91%)	
Type of surgery			NA
Debridement	99 (85.34%)	95 (87.16%)	
Debridement + ovarian cystectomy	10 (8.62%)	5 (4.59%)	
Debridement + myomectomy	3 (2.59%)	2 (1.83%)	
Debridement + bilateral tubal ligation	2 (1.72%)	6 (5.50%)	
Debridement + myomectomy + ligation	1 (0.86%)	1 (0.92%)	
Debridement + tubal repair and orthopedic	1 (0.86%)	0	
Pregnancy position			0.576
Oviduct	113 (97.41%)	104 (95.41%)	
Cornual	3 (2.59%)	4 (3.67)	
Abdominal cavity	0	1 (0.92%)	
Gestation (weeks)	7 (4, 10)	7 (4, 10)	0.056
ASA grading			0.085
II	57 (49.14%)	68 (62.39%)	
III	51 (43.97%)	38 (34.86%)	
IV	8 (6.90%)	3 (2.75%)	

NA: nonapplicable.

**Table 2:** Operative data.

Parameters	ICS (*n* = 116)	Controls (*n* = 109)	*P* value
Operation time (min)	81.5 (38, 190)	80 (35, 222)	0.393
Hospitalization days (days)	5 (2, 9)	5 (3, 9)	0.007
Cure rate	116 (100%)	109 (100%)	NA
Amount of blood loss (mL)	2020 (1210, 4500)	2000 (1200, 5000)	0.105
Amount of autologous transfusion (mL)	1500 (750, 3000)	NA	
Allogenic transfusion rate	3 (2.59%)	73 (66.97%)	<0.001
Infusion of allogenic coagulation factors	9 (7.76%)	46 (42.20%)	<0.001
ICU occupancy	0	1 (0.9%)	0.484
Classification of wound healing			NA
First intention	116 (100%)	109 (100%)	
Second intention	0	0	
Surgical complications			NA
Infection	0	0	
Wound bleeding	0	0	
Transfusion-related adverse reactions			NA
Allergy	0	0	
Purpura	0	0	
Nonhemolytic febrile reaction	0	0	
Bleeding	0	0	
Hemolysis	0	0	
Transfusion-related acute lung injury	0	0	
Anemia-related complications			NA
Dizziness	0	0	
Palpitation	0	0	
Chest distress and chest pain	0	0	
Hb at discharge (g/L)	87 (58, 110)	79 (54, 102)	<0.001

**Table 3:** Coagulation function.

	ICS (*n* = 116)			Controls (*n* = 109)			*P*	*P*
	Preoperative	Postoperative	*P*	Preoperative	Postoperative	*P*	(preoperative, ICS versus controls)	(postoperative, ICS versus controls)
Hb (g/L)	93.0 (39.0, 131.0)	87.0 (58.0, 110.0)^*∗*^	0.002	95.0 (37.0, 129.0)	79.0 (54.0, 102.0)^*∗*^	<0.001	0.681	<0.001
PT (s)	14 (11.1, 21.9)	14.3 (11.1, 20)^*∗∗*^	0.236	13.9 (12, 28.6)	14.1 (10.2, 21.6)^*∗∗*^	0.101	0.971	0.077
PT-INR	1.08 (0.91, 1.86)	1.10 (0.85, 1.68)^*∗∗*^	0.352	1.07 (0.87, 2.58)	1.07 (0.88, 1.85)^*∗∗*^	0.724	0.892	0.171
APTT (s)	33.5 (22.3, 49.4)	35.05 (20.4, 48.9)^*∗∗*^	<0.001	32.9 (22.4, 53.4)	33.7 (23.8, 50.2)^*∗∗*^	0.045	0.738	0.307
FIB (g/L)	2.46 (0.95, 6.10)	2.4 (0.76, 6.1)^*∗∗*^	0.285	2.53 (0.72, 6.10)	2.35 (1.09, 4.42)^*∗∗*^	0.222	0.509	0.993
TT (s)	16.3 (13, 21.8)	15.7 (11.7, 22.6)^*∗∗*^	0.002	15.5 (13.3, 21.9)	15.3 (11.5, 26.0)^*∗∗*^	0.63	<0.001	0.299

Hb: hemoglobin; PT: prothrombin time; APTT: activated partial thromboplastin time; FIB: fibrinogen; TT: thrombin time. ^*∗*^At discharge. ^*∗∗*^24 h after surgery.

**Table 4:** Characteristics of the patients according to the amount of blood loss.

	Blood loss (mL)	ICS (*n* = 116)	Controls (*n* = 109)	*P* value
*n*	1200–1599	36	43	0.796
	1600–1999	14	11	
	2000–2399	37	30	
	2400–2799	18	13	
	2800–3199	7	8	
	≥3200	4	4	

Requirement for allogenic blood (cases)	1200–1599	0	13 (30.23%)	<0.001
	1600–1999	1 (7.14%)	10 (90.91%)	<0.001
	2000–2399	1 (2.70%)	25 (83.33%)	<0.001
	2400–2799	1 (5.56%)	13 (100%)	<0.001
	2800–3199	0	8 (100%)	<0.001
	≥3200	0	4 (100%)	0.029

Requirement for coagulation factors (cases)	1200–1599	1 (2.78%)	5 (11.63%)	0.212
	1600–1999	1 (7.14%)	7 (63.64%)	0.007
	2000–2399	2 (5.41%)	15 (50.00%)	<0.001
	2400–2799	2 (11.11%)	9 (69.23%)	0.002
	2800–3199	2 (28.57%)	7 (87.50%)	0.041
	≥3200	1 (25.00%)	3 (75.00%)	0.486

Hemoglobin before discharge (g/L)	1200–1599	79.5 (59, 105)	82 (57, 96)	0.969
	1600–1999	91.5 (73, 109)	77 (60, 89)	0.004
	2000–2399	86 (65, 108)	74 (62, 102)	<0.001
	2400–2799	92.5 (58, 110)	76 (66, 92)	0.001
	2800–3199	81 (73, 93)	81.5 (54, 100)	0.955
	≥3200	73 (66, 91)	88.5 (63, 101)	0.57

**Table 5:** Subgroup analysis of coagulation function.

	Blood loss (mL)	ICS (*n* = 116)	Controls (*n* = 109)	*P* (ICS versus controls)
PT-pre (s)	1200–1599	13.85 (12.4, 21.7)	13.9 (12, 17.5)	0.655
	1600–1999	14.05 (12.7, 15.6)	14 (12.7, 15)	0.851
	2000–2399	14.1 (12.3, 21.9)	13.9 (12.2, 16.9)	0.328
	2400–2799	13.5 (11.1, 18.4)	13.7 (12.3, 16.6)	0.890
	2800–3199	13.0 (12.4, 14.7)	13.7 (12.9, 15.2)	0.189
	≥3200	14.2 (12.7, 14.6)	15.65 (13, 28.6)	0.200

PT-post (s)	1200–1599	14.7 (11.7, 20.0)	13.9 (10.3, 20)	0.033
	1600–1999	13.8 (12.5, 19.0)	15.3 (10.2, 16.3)	0.767
	2000–2399	14.3 (11.2, 19.8)	13.85 (10.7, 17.2)	0.097
	2400–2799	13.6 (12.3, 19.9)	13.8 (10.8, 16.3)	0.828
	2800–3199	14.0 (11.1, 15.3)	14.05 (11.3, 16.3)	0.955
	≥3200	14.2 (12.8, 15.2)	14.35 (13.2, 21.6)	0.686

PT-INR-pre	1200–1599	1.07 (0.96, 1.85)^*∗*^	1.07 (0.87, 1.43)	0.661
	1600–1999	1.12 (0.93, 1.25)	1.09 (0.95, 1.17)	0.403
	2000–2399	1.09 (0.91, 1.86)	1.07 (0.89, 1.35)	0.492
	2400–2799	1.06 (0.93, 1.54)	1.04 (0.9, 1.35)	1.000
	2800–3199	0.99 (0.96, 1.15)	1.08 (0.98, 1.15)	0.121
	≥3200	1.11 (0.95, 1.14)	1.23 (0.97, 1.58)	0.200

PT-INR-post	1200–1599	1.14 (0.95, 1.68)^*∗*^	1.07 (0.88, 1.56)	0.009
	1600–1999	1.07 (0.91, 1.57)	1.19 (0.92, 1.29)	0.609
	2000–2399	1.12 (0.91, 1.65)	1.07 (0.9, 1.38)	0.434
	2400–2799	1.02 (0.9, 1.67)	1.04 (0.89, 1.29)	1.000
	2800–3199	1.07 (0.85, 1.23)	1.06 (0.9, 1.29)	0.955
	≥3200	1.08 (0.94, 1.21)	1.09 (0.98, 1.85)	0.686

APTT-pre (s)	1200–1599	33.7 (23.6, 49.4)^*∗∗*^	33.4 (23.2, 45.4)	0.848
	1600–1999	33.8 (29, 48.6)	33.2 (26.6, 45)^#^	0.851
	2000–2399	33.6 (26.3, 44.6)	32.2 (22.4, 46.8)	0.304
	2400–2799	31.9 (23.6, 49.4)	32.8 (26.8, 39.8)	0.293
	2800–3199	33.0 (22.3, 37.9)	33.6 (31.6, 39.8)	0.779
	≥3200	33.0 (31.7, 40.5)	30.7 (29, 53.4)	0.486

APTT-post (s)	1200–1599	35.8 (28.1, 45.1)^*∗∗*^	34.2 (23.8, 49.1)	0.236
	1600–1999	35.2 (31.2, 48.9)	36.2 (24.3, 48.1)^#^	0.936
	2000–2399	34.2 (28.8, 47.5)	33.2 (27.3, 42.8)	0.287
	2400–2799	33.9 (27.4, 44.7)	33.6 (26.8, 44.3)	0.650
	2800–3199	37.0 (20.4, 38.8)	35.2 (30.2, 44.2)	0.779
	≥3200	33.8 (29.3, 44.2)	35.5 (28.7, 50.2)	1.000

FIB-pre (g/L)	1200–1599	2.49 (0.95, 4.28)	2.31 (0.72, 6.10)	0.497
	1600–1999	2.24 (1.59, 3.95)	2.69 (2.12, 4.17)	0.107
	2000–2399	2.51 (1.39, 4.83)	2.67 (1.51, 3.93)	0.801
	2400–2799	2.36 (1.31, 3.79)	2.64 (1.42, 3.88)	0.051
	2800–3199	2.46 (1.81, 3.47)	2.67 (2.34, 4.14)	0.397
	≥3200	2.54 (2.21, 2.84)	2.05 (0.73, 2.90)	0.486

FIB-post (g/L)	1200–1599	2.37 (0.76, 3.81)	2.33 (1.09, 4.42)	0.918
	1600–1999	2.20 (1.18, 3.33)	2.31 (1.10, 3.45)	0.979
	2000–2399	2.58 (1.21, 3.69)	2.5 (1.45, 3.78)	0.753
	2400–2799	2.29 (1.07, 4.2)	2.42 (1.75, 3.56)	0.489
	2800–3199	2.58 (1.90, 3.54)	2.31 (2.03, 3.02)	0.779
	≥3200	2.56 (1.62, 3.65)	2.12 (1.65, 3.65)	0.686

TT-pre (s)	1200–1599	16.6 (13.5, 21.8)	16.0 (13.3, 21.9)	0.128
	1600–1999	15.9 (14.3, 17.7)	15.5 (13.7, 18.8)	0.501
	2000–2399	16.2 (13.7, 21.8)	15.1 (13.5, 17.1)	<0.001
	2400–2799	16.4 (14.3, 20.3)	15.4 (14.5, 19)	0.089
	2800–3199	17.2 (13, 18.7)	15.1 (13.7, 17.1)	0.054
	≥3200	16.7 (14.7, 19)	15.6 (14.7, 20.3)	0.886

TT-post (s)	1200–1599	15.6 (12.7, 22.6)	15.3 (13.1, 26)	0.690
	1600–1999	15.3 (13.5, 17.8)	17.2 (13.7, 17.9)	0.647
	2000–2399	16.3 (13.2, 19.6)	15.2 (11.5, 17.8)	0.001
	2400–2799	15.6 (13.1, 20.6)	15.1 (12.4, 17.6)	0.312
	2800–3199	13.9 (11.7, 18.3)	15.3 (14.6, 19.7)	0.072
	≥3200	15.9 (15.2, 17.8)	16.5 (14.2, 18.9)	1.000

^*∗*^ *P* = 0.032, ^*∗∗*^*P* = 0.017, and ^#^*P* = 0.050 between the two pre/post values.
